# Soft Sensory-Motor System Based on Ionic Solution for Robotic Applications

**DOI:** 10.3390/s24092900

**Published:** 2024-05-01

**Authors:** Sender Rocha dos Santos, Eric Rohmer

**Affiliations:** DCA-FEEC-UNICAMP, Sao Paulo 13083-852, Brazil

**Keywords:** sensory-motor, morphologic computation, soft sensors, actuators, soft robot applications, soft robot materials, designs

## Abstract

Soft robots claim the architecture of actuators, sensors, and computation demands with their soft bodies by obtaining fast responses and adapting to the environment. Sensory-motor coordination is one of the main design principles utilized for soft robots because it allows the capability to sense and actuate mutually in the environment, thereby achieving rapid response performance. This work intends to study the response for a system that presents coupled actuation and sensing functions simultaneously and is integrated in an arbitrary elastic structure with ionic conduction elements, called as soft sensory-motor system based on ionic solution (SSMS-IS). This study provides a comparative analysis of the performance of SSMS-IS prototypes with three diverse designs: toroidal, semi-toroidal, and rectangular geometries, based on a series of performance experiments, such as sensitivity, drift, and durability. The design with the best performance was the rectangular SSMS-IS using silicon rubber RPRO20 for both internal and external pressures applied in the system. Moreover, this work explores the performance of a bioinspired soft robot using rectangular SSMS-IS elements integrated in its body. Further, it investigated the feasibility of the robot to adapt its morphology online for environment variability, responding to external stimuli from the environment with different levels of stiffness and damping.

## 1. Introduction

In recent years, the robotic application has been investigating new soft materials for integration in the robot systems bringing flexibility and best results compared with the traditional rigid body robots. In this way, soft robots claim an architecture of actuators, sensors, and computation demand with their soft bodies by obtaining fast responses, adapting to the environment, and undergoing high deformations in interaction [1,2,3]. Sensory-motor coordination is one of the main design principles acquired by soft robots for the capability of mutual coupling of sensing and acting, allowing the mechanical systems to receive fast mechanical feedback from the task environment, triggering an internal physical stimulation to the sensory system, and in combination with the external physical stimulation from the sensory receptors, it can provide a sensory feedback to the controller more accurately and rapid [4,5,6].

The Force Sensing Resistors (FSRs) are devices that allow measuring static and/or dynamic forces applied on a surface where it is placed, through the variation in its electric resistance [7]. Several kinds of soft sensors have been developed for robotic applications based on the FSR principle, such as EGaIn [8] and ConTact Sensor [9]. The FSR sensors fit well to the sensory-motor element for soft robots because their body can show a good deformability performance, i.e., acting actively in the environment and sensoring passively the external stimuli. 

To achieve a soft sensory-motor system with the characteristics of expansion and compression, ionic soft sensors were investigated. Several ionic soft sensors have been developed [10,11,12]. Recent research has shown that ionic resistance ionic results in easy fabrication and good performance. 

The ionic solution used in the sensory-motor system is composed of the following elements: aloe vera, glycerol, and salt. The elements present in the solution allow the solution to have a fast ion conductor performance. As shown in [13], there are many chemical constituents in aloe vera including inorganic compounds such as Calcium, Chlorine, Chromium, Copper, Iron, Magnesium, Manganese, Potassium, Phosphorous, Sodium, and Zinc. Also, as shown in [14], aloe vera leaf has a strong electrical anisotropy. In all directions across the conductive bundle, the behavior of the system is completely passive and linear like in a regular electric circuit with a constant resistance. Aloe is the main component for supplying cations and anions to the solution, and therefore, it is the component with a high concentration of carrier charges, allowing the solution to be a fast ion conductor. 

As shown in [15,16], glycerol also enhances the ionic conductivity. From [17], it can be inferred that conductivity increases dramatically for most glycerin–water mixture ratios. Therefore, glycerin contributes to the conductivity medium of the solution. Since aloe is a quasi-solid-state solution that is associated with difficulty in ion transportation between electrodes, glycerin reduces the viscosity of the aloe, improving the ion mobility in the solution.

NaCl is added to the solution to provide additional cations and anions for ionic redistribution in the ionic solution [17]. Therefore, the elements present in the solution allow the solution to have a fast ion conductor performance. 

The performance results of the soft sensors using ionic solutions are influenced by several factors, including their working principles, sensing material properties, geometries, etc. [18,19]. Based on [17], it is shown that different geometries of external pressure sensors present different performance results such as sensibility, durability, and drift, meaning that the design can influence the performance results of these classes of sensors. Furthermore, the morphology of a soft actuator is important for making a control system for a soft robot. This is because, unlike a hard robot, a soft robot can change volume and shape based on pneumatic or hydraulic inflation pressure or by forces in the external environment. In addition, unlike a hard robot, the response of the soft material of the actuator to force, whether external or internal, is highly non-linear, making calculations that predict the behavior of the actuator in response to force very complex and difficult [20]. This work intends to provide a comparative study of the performance regarding different designs of soft sensory-motor system based on ionic solution (SSMS-IS), i.e., a system able to sense external and internal pressures and to actuate in the environment using the same soft body. 

Robots based on morphological computation have a good match according to the SSMS-IS working principle once the input is received directly through the body by interaction with the environment including other agents in the form of forces. The robot body serves as an actuator (as an output, it applies forces) as well as a sensor (reacts physically to input forces) [21]. 

Therefore, the main goal of this work was to create a unique system interface capable of sensing and acting simultaneously. In other words, the robot shall present two states of behavior: in the passive mode, it can perceive the environment as a sensor, and in the active mode, it can actuate in the environment as an actuator. 

The SSMS-IS integrated into the robot body allows the robot to adapt its morphology online for environment variability, responding to external stimuli from the environment with different levels of stiffness and damping. For example, the robot body can detect risks in the environment, such as external input forces that might damage the system, and, simultaneously, the segments can be actuated to reduce the impact of external forces such as a mechanism of self-protection. 

This study aims to investigate the evidence of the performance deviation based on three different geometries of the sensory-motor system based on the same ionic solution, and to answer the following question: can we have different performances for different designs of soft sensory-motor systems based on ionic solution (SSMS-ISs) for the same test conditions? Further, this study intends to investigate if the best design of SSMS-IS can perform a morphologic computation in the soft robot body for the same test conditions.

This manuscript is organized as follows: Section 2 explains our proposed sensor design and fabrication and introduces the design parameters considered for the optimization problem. Section 3 describes the characterization experiments performed on the prototypes and discusses the results to identify the pros and cons of each solution. Section 4 provides a summary of the work performed. 

## 2. Materials and Methods

**Figure 1:**
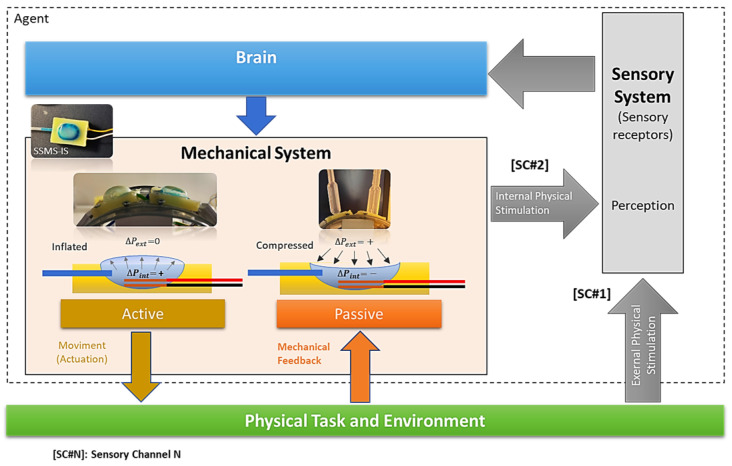
Diagram of the robot principle: the system designed can perceive the environment as a sensor and actuate in the environment as an actuator using an integrated soft body. The internal mechanical feedback from the body can be combined with external physical stimulation representing a redundancy of the sensory channels, improving the speed and simplifying the perception. The information processed by the perception module can go to the brain and activate the actuator part of the system, but it also can be processed in the sensory-motor coordination module without necessarily passing through the brain, speeding up the dynamic control of the robot.

The design of the robot’s shield in this work was bioinspired and was based on the morphology of the terrestrial isopod Armadilium Vulgare since its shape has multi-purpose advantages such as protecting its inner area during conglobation (rolling-up behavior) as a mechanism of self-protection [22,23,24]. Each segment of the soft robot should have a sensory-motor based on the soft sensory-motor system based on ionic solution (SSMS-IS) to actuate in the environment when expanded and to sense when compressed. As shown in Figure 1, both states integrated into only one component allow the system performance in a sensory-motor coordination mode, i.e., in a dynamic and reciprocal coupling among the brain (control), the body, and the environment [25].

### 2.1. SSMS-IS Design

**Figure 2:**
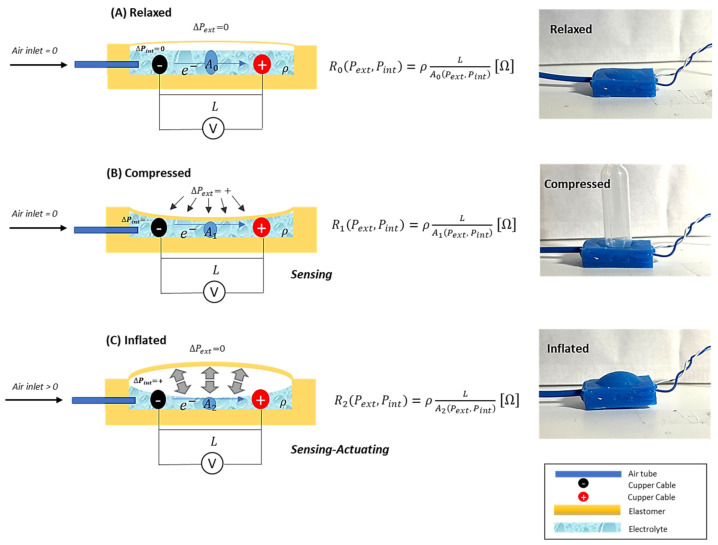
Diagram of the principle of functionality regarding soft sensory-motor system based on ionic solution. Three states of the SSMS-IS can be observed, i.e., (**A**) relaxed, when there is no air inlet in the system $\left(\Delta P_{int}=0\right)$ and the external pressure is null $\left(\Delta P_{ext}=0\right)$; (**B**) compressed, when there is no air inlet in the system $\left(\Delta P_{int}=0\right)$ and there is an external pressure $\left(\Delta P_{ext}\neq 0\right)$ in the system; and (**C**) inflated, when there is air inlet $\left(\Delta P_{int}\neq 0\right)$ in the system and the external pressure is null $\left(\Delta P_{ext}=0\right)$. For each state, differences can be observed in the electric resistance measurement $\left(R_{x}\right)$ for a determined voltage (V) based on the variation in the ionic solution area $\left(A_{x}\right)$ and distance (L) between the positive and negative cupper cable. The variation in the ionic solution area $\left(A_{x}\right)$ is correlated with the variation in external pressure $\left(\Delta P_{ext}\right)$ and internal pressure $\left(\Delta P_{int}\right)$.

As shown in Figure 2, there are 3 states of the system: relaxed, inflated, and compressed. In the relaxed state, there is no air inlet in the sensor, and the variation in the internal and external pressures is null. In the inflated state, there is an air inlet in the sensor, and the internal pressure increases making the sensor inflated, once the body is deformable. For the compressed state, the air inlet is null as well, but an external pressure is applied to the sensor, making the sensor compressed. For each state, the electrolyte in the sensor varies its volume, consequently changing its internal electric resistance. 

**Figure 3:**
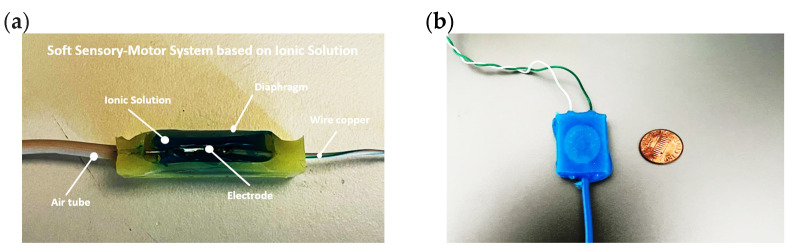
(**a**) Internal components of soft sensory-motor system based on ionic solution: ionic solution, soft diaphragm, copper wire, air tube, and electrode. The internal view is based on the rectangular geometry but can be applied to any design of SSMS-IS. (**b**) Comparison of the size of the SSMS-IS rectangular design with a 1 cent coin.

As shown in Figure 3, the SSMS-IS is composed of an ionic solution, a soft diaphragm to allow the expansion and compression of the system; two copper wire to conduct the electric current in the electrolyte; two metal electrodes submerged in the ionic solution to guarantee the electric current conduction; and an air tube to allow the inlet of air to guarantee the inflation of the system. 

### 2.2. Robot Design

**Figure 4:**
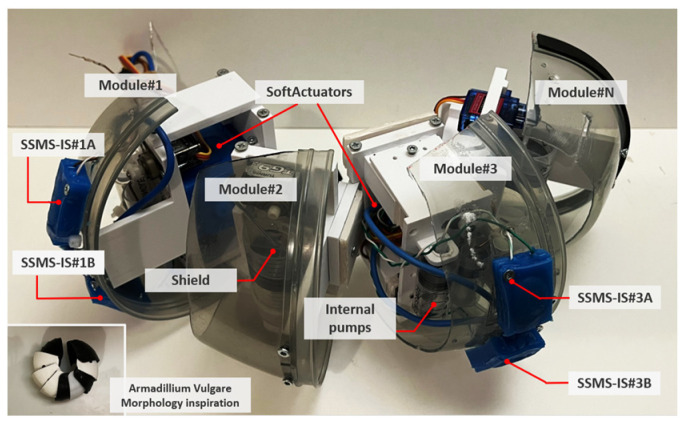
Bioinspired robot design based on the morphology of the isopod Armadilium Vulgare: it was formed by modules that represent the bioinspired segments and each segment of the soft robot should have a sensory motor based on the SSMS-IS to actuate in the environment when expanded and to sense when compressed. Also, it can execute the conglobation movement for self-protection using soft actuators in each segment.

**Figure 5:**
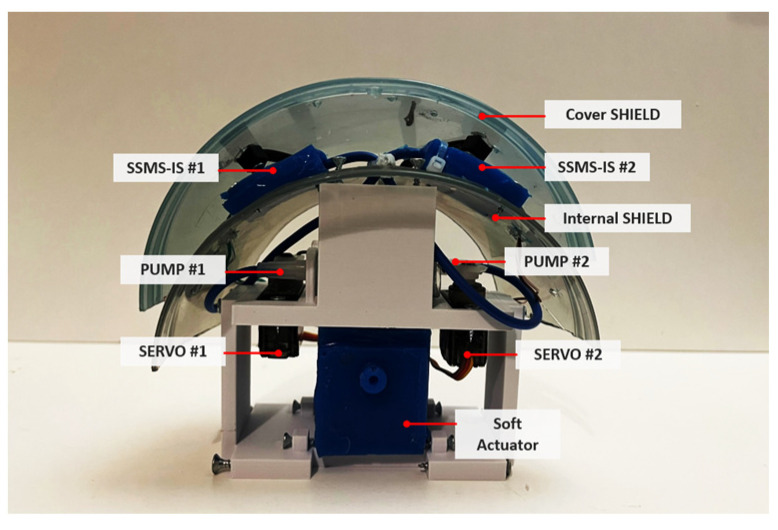
Details of the robot’s segment with two SSMS-IS elements integrated into an internal interface. Each segment is composed of two pumps to inflate the SSMS-IS, two servo motors to make the pumps made by syringes function, and a soft actuator responsible for generating the conglobation movement bioinspired from Armadillium Vulgare. The SSMS-IS elements are actuated by pumps embedded in the robot.

The design of the robot’s shield was bioinspired and was based on the morphology of the terrestrial isopod Armadilium Vulgare, which has a total of 8 segments, allowing the execution of a coordinated movement during conglobation. Based on this, as shown in Figure 4 and Figure 5, the proposed system was formed by modules that represent the bioinspired segments, and the robot body incorporates two rectangular SSMS-ISs mounted on the surface shield. Bioinspired robot design based on the morphology of the isopod Armadilium Vulgare: it was formed by modules that represent the bioinspired segments and each segment of the soft robot should have a sensory motor based on the SSMS-IS to actuate in the environment when expanded and to sense when compressed. Also, it can execute the conglobation movement for self-protection using soft actuators in each segment.

On each side of the concave shield of each robot module, one rectangular SSMS-IS was positioned to detect internal and external pressures that counter the interface robot. The determination of the sensor’s position in the shield was investigated based on simulation using the CoppeliaSim Edu software, version 4.5.1 [26]. Then, with 2 sensors per module, there are 4 possible states of actuation, as shown in Table 1. 

**Figure 6:**
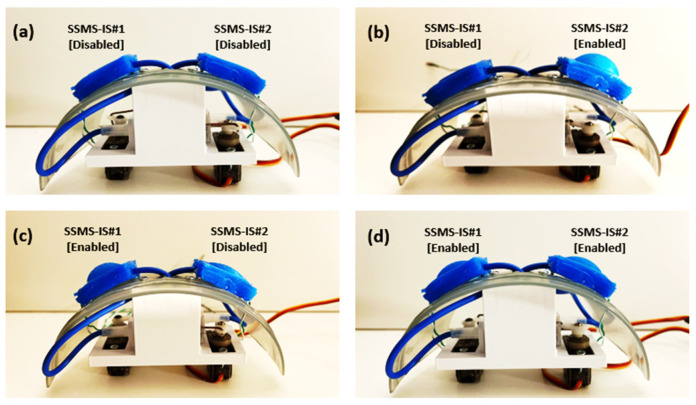
Robot morphology adaptation. From Table 1, (**a**) State 1, when both SSMS-ISs in the soft robot shield are disabled; (**b**) State 2, when one SSMS-IS is enabled; (**c**) State 3, when one SSMS-IS is enabled; and (**d**) State 4, when both SSMS-ISs are enabled.

Consequently, as shown in Figure 6, when both SSMS-ISs are enabled, the soft robot body can adapt both sides of its morphology, and when only one system is enabled, the body can adapt only one side of its morphology. When both systems are disabled, then the soft robot body can only sense the external forces from the environment through its body. 

### 2.3. Fabrication

The sensor prototypes were manufactured with two main components, the elastomer material that deforms the sensor body in response to internal and external pressures and the ionic sensing gel. The elastomer used was silicon rubber RPRO 20 (Reschimica^®^, Florence, Italy) mixed in a 1:1 ratio by weight. The ionic solution was made by mixing 40.00% of glycerin, 54.64% of aloe vera (99% composition solution), and 5.46% of NaCl (domestic salt). A liquid dye color is also added to make the manipulation easy during the sensor fabrication since the ionic solution is transparent. To further investigate the performance of the system’s deformability (compression and expansion), an alteration in the geometry sensors was investigated. 

**Figure 7:**
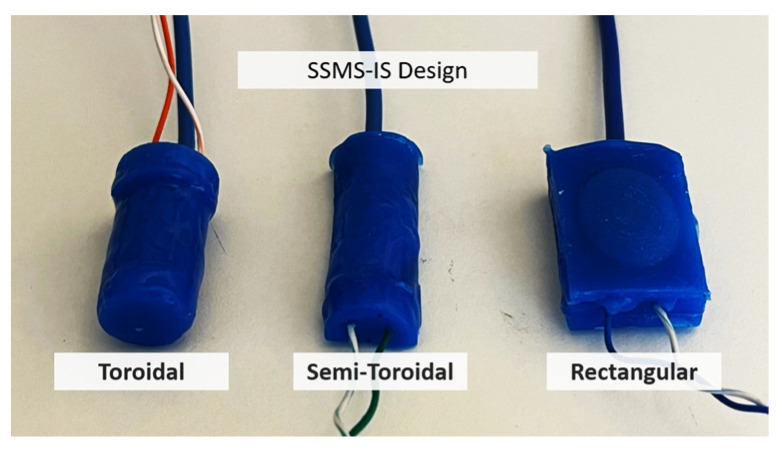
Three SSMS-IS designs were investigated: toroidal, semi-toroidal, and rectangular geometries. They were fabricated using silicon rubber RPRO 20 (Reschimica^®^).

Three different molds were made based on the different geometries: toroidal, semi-toroidal, and rectangular, as shown in Figure 7. The toroidal SSMS-IS forms a soft cylindrical container with 7 mm outer radius and 27 mm length. The semi-toroidal SSMS-IS forms a soft half-cylindrical container with 6.93 mm outer radius and 38 mm length. The rectangular SSMS-IS forms a soft rectangular container with a height of 6 mm, a width of 20 mm, and a height of 30 mm. 

**Figure 8:**
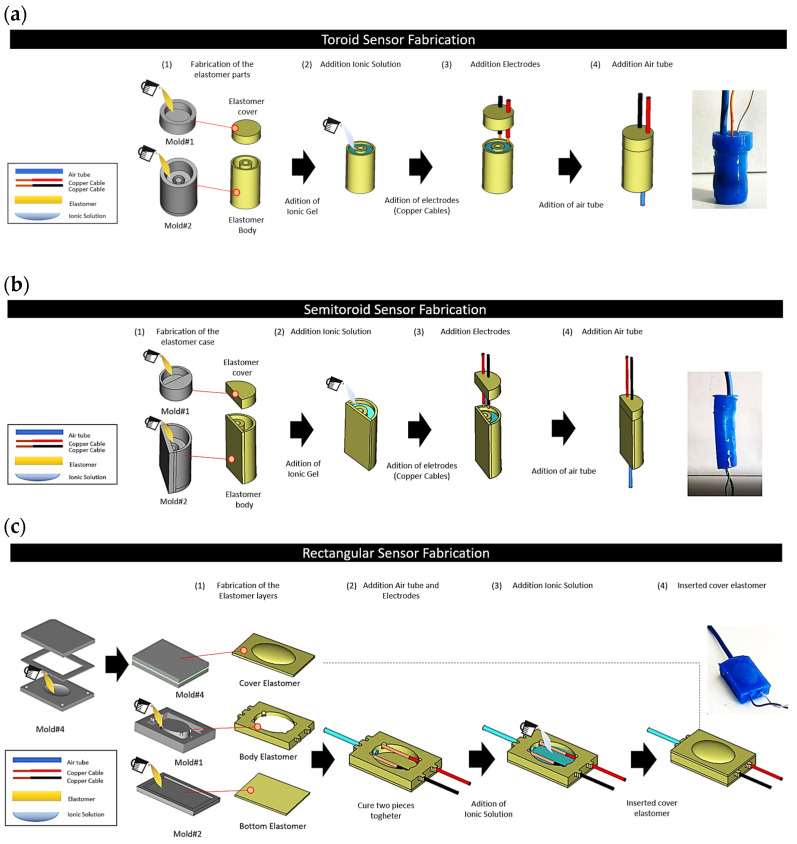
Fabrication procedure for different SSMS-IS designs: (**a**) SSMS-IS manufacturing process for toroid geometry; (**b**) SSMS-IS manufacturing process for semi-toroid geometry; and (**c**) SSMS-IS manufacturing process for rectangular geometry.

Figure 8 shows the SSMS-IS fabrication procedure for each proposed design. The silicon rubber component was cured in each 3D-printed mold, and the ionic conductive solution was filled into each prototype. Afterwards, copper wires were inserted into the cover body to form an electrical connection. Also, a thin flexible tube of 2 mm in diameter was inserted in each prototype to allow the air inlet in the sensor. At this point, the main container bodies were sealed with the cover layer using the uncured silicon rubber as a glue, resulting in a layer to seal the air inside the system. 

**Figure 9:**
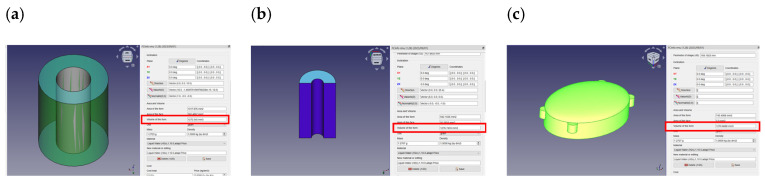
Design of the electrolyte volume for each mold: (**a**) toroidal electrolyte volume = 1272.35 mm^3^; (**b**) semi-toroidal electrolyte volume = 1276.74 mm^3^; and (**c**) rectangular electrolyte volume = 1270.67 mm^3^.

As shown in Figure 9, to guarantee the same comparison between the three soft motor sensors, each mold was designed with the same volume of electrolyte equal to 1.27 mL. The calculation was performed in the FreeCad software, version 0.20.2, using the macro FCInfo. 

**Figure 10:**
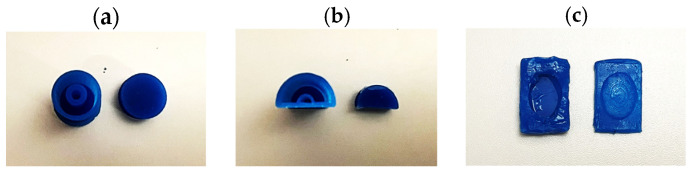
Elastomer parts for each SSMS-IS design: (**a**) toroidal; (**b**) semi-toroidal; and (**c**) rectangular. The diaphragm’s thickness for each SSMS-IS was 1 mm to guarantee less resistance during mechanical expansion.

The sensors were designed to have the same thickness in the external radius for the toroidal and semi-toroidal geometries and for the diaphragm of the rectangular geometry. The diaphragm’s thickness for each SSMS-IS was 1 mm to guarantee less resistance during mechanical expansion, as shown in Figure 10. But depending on the application, the thickness can be changed to obtain another sensor–motor performance such as robustness.

**Figure 11:**
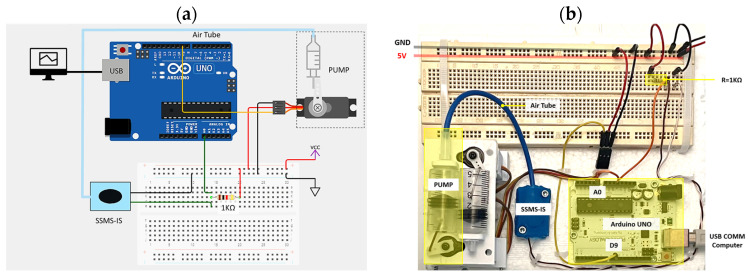
(**a**) Circuit diagram regarding the acquisition data for the resistance variation in the SSMS-IS. (**b**) Setup of the tests composed of controllers, pump, resistance divider circuit, SSMS-IS, and battery.

The resistance changes in the SSMS-IS were measured using a voltage divider, and the measures were monitored by the microcontroller Arduino Uno, reading the analog serial values. For the voltage divider, a resistor of 1 KΩ and a supplier voltage of 5 Vcc were used. The temperature condition of the tests was room temperature around 25 °C. The setup of tests was built using a protoboard, one air pump made using a servo motor, and a syringe of 10 mL. The air tube connected to the pump inflates the SSMS-IS. One wire cable of the SSMS-IS was connected to the ground and the other one was connected to the resistance. The connection between SSMS-IS and the resistance was connected with Arduino in the analog pin A0. The servo motor was connected to the D9 Arduino digital pin, as shown in Figure 11. The Arduino was connected to the computer by a USB cable. Also, the microcontroller controls the servo motor that is responsible for actuating the air pumps that have the function of inflating the SSMS-IS when requested. The acquisition data of the resistance variation over time was acquired by the software MATLAB, version 2022b, Support Package for Arduino Hardware.

**Figure 12:**
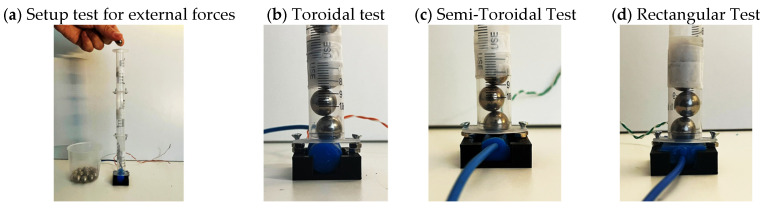
(**a**) Test setup for evaluating external pressure over different designs of SSMS-IS; (**b**) test setup for toroidal SSMS-IS; (**c**) test setup for semi-toroidal SSMS-IS; and (**d**) test setup for rectangular SSMS-IS.

The internal pressure input for the sensors was applied using a conventional syringe of 10 mL of total volume applying a positive pressure over the SSMS-IS when moving the syringe piston for each 1 mL volume for a specific time interval. The external input for the sensors was applied using a setup with a vertical tube of 30 cm exercising specific compression force when adding balls of mass that are 8.4 g each for a specific time interval over the SSMS-IS, as shown in Figure 12. The step response of the sensor was recorded by applying pressure in incremental steps of 4.6 mmHg. The next experiment subjected the SSMS-IS to repeated cycles of internal pressure of 224.61 mmHg. To evaluate the performance regarding drift, each SSMS-IS design was tested without load for a prolonged duration of 15 min to observe some drift in the system. To guarantee a reasonable comparison among designs, all tested SSMS-IS designs used the same concentration of ionic solution and the same quantity of the electrolyte. 

**Figure 13:**
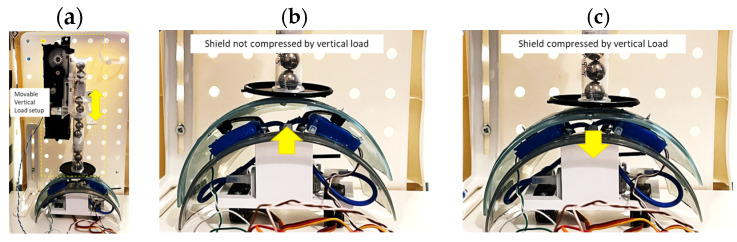
(**a**) Setup to test the response of both sensors simultaneously on the interface for external force input in the vertical direction as shown by the yellow arrow; (**b**) robot without external input force; and (**c**) vertical load pressing the shield in the vertical direction as shown by the yellow arrow in the picture, emulating an external input force over the robot.

The robot test was executed using a movable vertical load that can compress the shield in the vertical direction, as shown in Figure 13a. The setup can regulate different values of loads, but in this investigation, only three approximate values of loads were tested, i.e., 1.8 N, 2.4 N, and 2.9 N. Both rectangular SSMS-ISs integrated into the interface have been pressed over a cover rigid shield with the function to distribute equally the forces applied by the movable vertical load over the SSMS-IS located at the interface, as shown in Figure 13b,c.

The test was composed of 2 main steps: when the shield was in the relaxed state and when the shield was in the actuated state. For each state, the robot was subjected to 3 values of loads. The SSMS-IS located at the interface was simultaneously actuated and relaxed.

**Figure 14:**
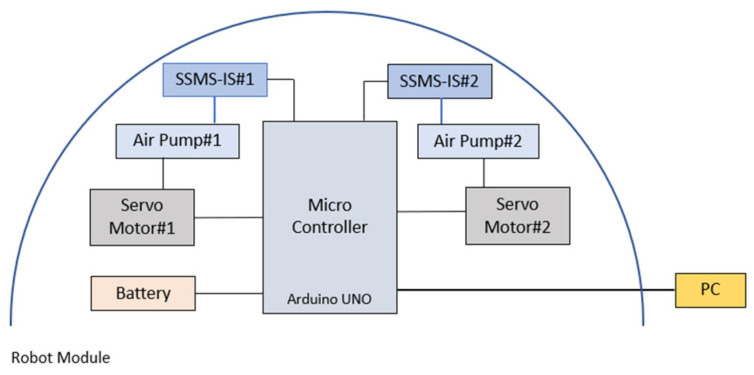
Robot module system configuration: one microcontroller; two SSMS-ISs; two servomotors; two air pumps connected with SSMS-IS; and a lithium-ion battery. The measurements performed by the microcontroller can be communicated with the PC.

Figure 14 presents the robot system configuration. The robot has a main microcontroller using Arduino Uno for reading the analog serial values from the SSMS-IS’s and controlling the servo motors that are responsible for actuating the air pumps. The air pumps have the function of inflating the SSMS-IS when requested. A lithium-ion battery supplied the controller and the acquisition data of the resistance variation over time for each SSMS-IS acquired by the software MATLAB Support Package for Arduino Hardware. 

## 3. Results

### 3.1. Performance for External Input Pressure of Different Designs of SSMS-IS

**Figure 15:**
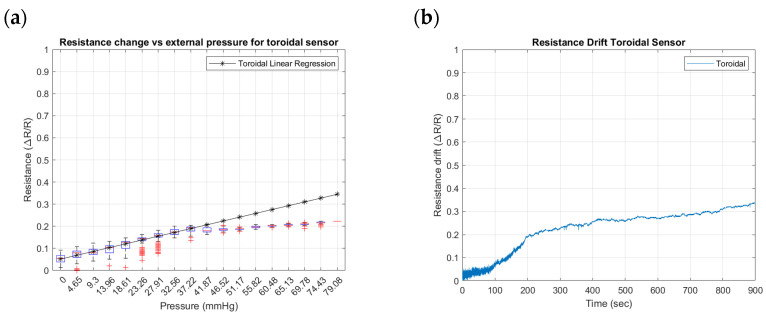
Resistance changes vs. pressure for toroidal geometry of the sensor: (**a**) sensitivity within the sensing range of external pressure of 0–79.08 mmHg and (**b**) drift in toroidal sensor for approximately 15 min.

**Figure 16:**
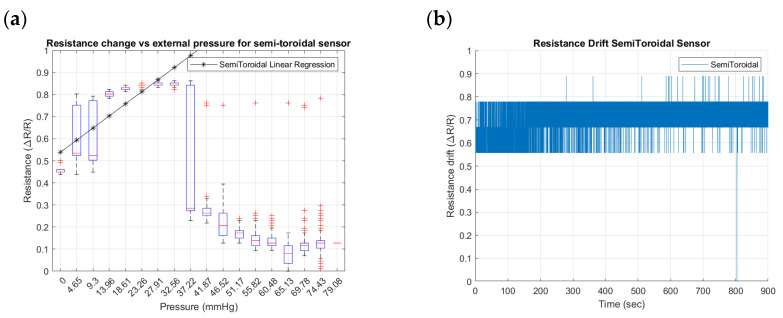
Resistance changes vs. pressure for semi-toroidal geometry of the sensor: (**a**) sensitivity within the sensing range of external pressure of 0–79.08 mmHg and (**b**) drift in semi-toroidal sensor for approximately 15 min.

**Figure 17:**
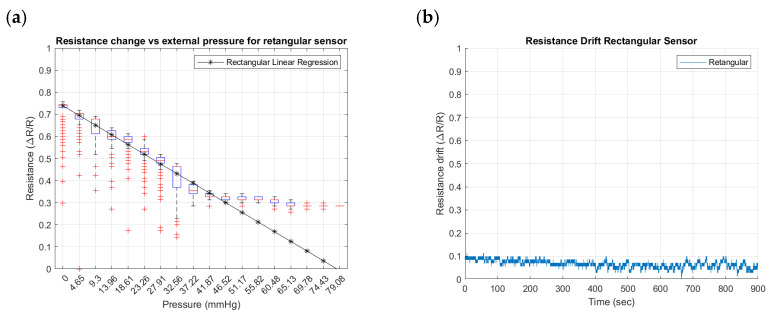
Resistance changes vs. pressure for the rectangular geometry of the sensor: (**a**) sensitivity within the sensing range of external pressure of 0–79.08 mmHg and (**b**) drift in rectangular sensor for approximately 15 min.

To identify the differences that geometry can induce in the performance of external pressure inputs in the SSMS-IS, three geometrical designs were tested for their sensitivity and drift: toroidal, semi-toroidal, and rectangular. Figure 15, Figure 16 and Figure 17 summarize the results from the experiments performed on the toroidal, semi-toroidal, and rectangular designs for external pressure, respectively. 

From Figure 15a, Figure 16a and Figure 17a, the rectangular design shows the highest sensitivity, as it has the highest gradient in the linear regression curve of resistance. There are more outlier values for low pressures because of the ball bouncing for the test setup, especially for the rectangular geometry. Also, a linear sensitivity can be noticed for the range of input pressures between 0 and 37.22 mmHg for all investigated SSMS-IS designs. 

Regarding the drift shown in Figure 15b, Figure 16b and Figure 17b, the rectangular design shows a lower drift for the same defined time. The drift in the semi-toroidal system, despite not increasing with time, shows a large variation in resistance in comparison to the toroidal and rectangular designs. 

### 3.2. Performance for Internal Input Pressure of Different Designs of SSMS-IS

**Figure 18:**
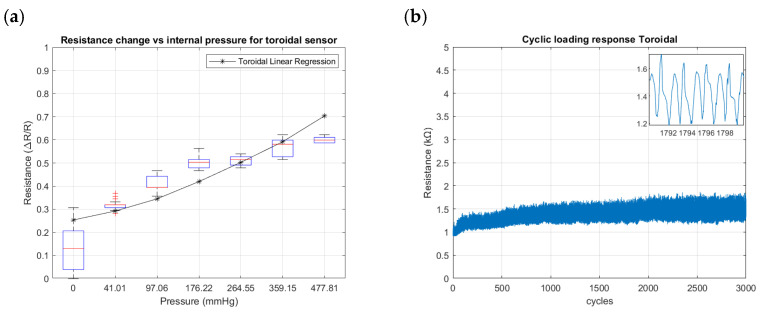
Resistance changes vs. internal pressure for toroidal geometry of the sensor: (**a**) sensitivity within the sensing range of external pressure of 0–477.81 mmHg and (**b**) results of cyclic loading of the sensor for 3000 cycles of internal pressure at 224.61 mmHg.

**Figure 19:**
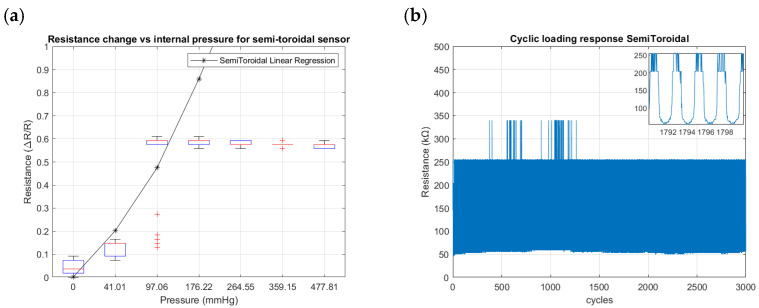
Resistance changes vs. internal pressure for semi-toroidal geometry of the sensor: (**a**) sensitivity within the sensing range of external pressure of 0–477.81 mmHg and (**b**) results of cyclic loading of the sensor for 3000 cycles of internal pressure at 224.61 mmHg.

**Figure 20:**
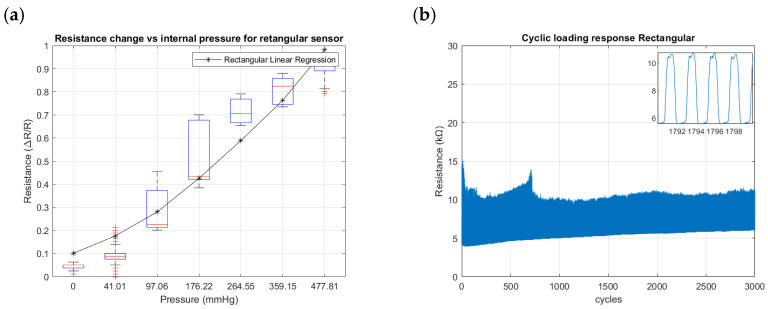
Resistance changes vs. internal pressure for rectangular geometry of the sensor: (**a**) sensitivity within the sensing range of external pressure of 0–477.81 mmHg and (**b**) results of cyclic loading of the sensor for 3000 cycles of internal pressure at 224.61 mmHg.

Figure 18, Figure 19 and Figure 20 summarize the results from the experiments performed on the toroidal, semi-toroidal, and rectangular SSMS-ISs for internal pressure input, respectively. 

From Figure 18a, Figure 19a and Figure 20a, the rectangular design shows the highest sensitivity, as it has the highest gradient of resistance. The semi-toroidal design shows a saturation for pressure values more than 97 mmHg. 

Regarding the cycle tests shown in Figure 18b, Figure 19b and Figure 20b, the SSMS-IS presents a constant level of durability. However, it is possible to note that the rectangular design shows the highest variation in resistance to internal pressure. 

### 3.3. Performance of SSMS-IS in the Robot Application

**Figure 21:**
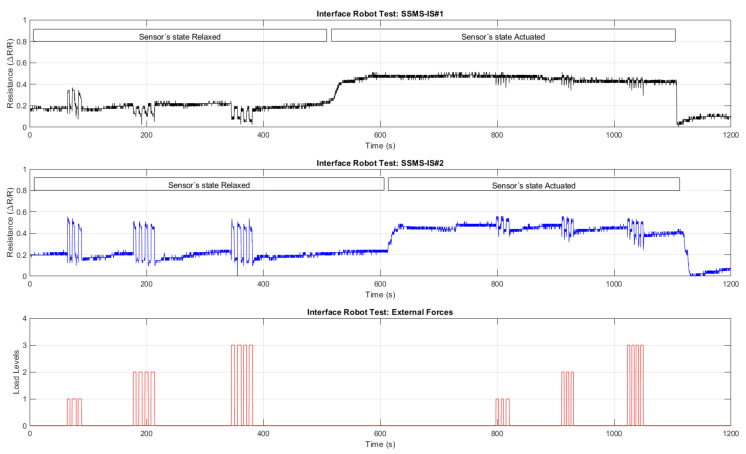
Robot time response for two states over external pressure inputs. Initially, the robot was in a relaxed state, and after subjecting the robot to 3 levels of loads (1.8 N, 2.4 N, and 2.9 N), the levels of force due to the variation in the electric resistance of the SSMS-IS was detected. In the second moment of the test, the robot was in the actuated state, and after subjecting the robot to 3 levels of loads (1.8 N, 2.4 N, and 2.9 N), the levels of force due to the variation in the electric resistance of the SSMS-IS was detected, indicating that, even in the actuated state, the robot can sense differences in external forces using a unique device to actuate and to sense simultaneously.

Figure 21 summarizes the result from the experiment performed on the interface robot integrated with two rectangular SSMS-ISs responding to external pressure inputs. Only the states in which both systems are enabled or disabled have been evaluated, i.e., the robot states 1 and 4 as in Table 1. Side pressure input directions were not within the scope of the tests. 

As shown in Figure 21, the robot performs in two types of states: relaxed and actuated. Three levels of force loads are applied over the interface for each robot state. The level of each force load is monitored for the variation in the resistance for each SSMS-IS in the shield for both robot states: relaxed and actuated. As shown in Figure 17a and Figure 20a, the variation in the SSMS-IS resistance has a linear response with the input pressure applied over and inside the SSMS-IS. Then, the robot response in Figure 21 indicates that the robot can monitor the impact of external forces in both states of the robot. 

**Figure 22:**
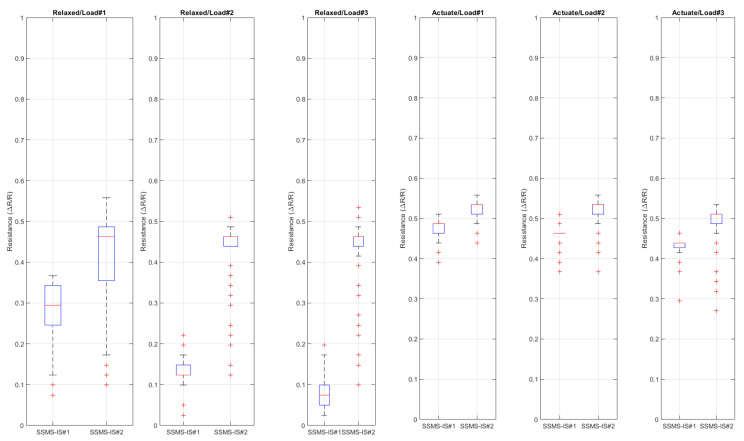
Boxplot for the soft robot response for each load applied in each performed state. For the relaxed state, each SSMS-IS in the robot could detect the external loads by the variation in the electric resistance. Also, when the value of the load was increased, the variation in the resistance proportionally increased. For the actuated state, each SSMS-IS in the robot could also detect the external loads by the variation in the electric resistance. When the value of the load was increased, the variation in the resistance also proportionally increased, indicating that in both states, relaxed and actuated, the robot can measure different external load values, and the variation in the internal resistance is proportional to the variation in the load.

Figure 22 shows the boxplot analysis of the robot time response provided in Figure 21. Each boxplot shows the resistance distribution for each SSMS-IS in the soft robot body regarding each set of inputs for each state of the robot. 

## 4. Discussion

From the test results, it can be inferred that it is possible to have a performance deviation based on different designs of the sensory-motor system based on the same ionic solution for the same test conditions.

The results show that different designs of SSMS-IS can have different sensitivity performances for the same test condition. As shown in Figure 15a, Figure 16a and Figure 17a, there are different levels of sensitivity for the percentage of linear variation and linear range for each design tested in the same variation of external pressure. For the toroidal design, a sensitivity variation of 20% was observed, while for the rectangular design, approximately 45% of sensitivity variation was observed, making it the sensor with the highest linear external sensitivity variation in comparison to the other studied designs. Also, from the linear regression in Figure 15a, Figure 16a and Figure 17a and Table 2, there are differences in the linear sensitivity range for external forces. The semi-toroidal design showed a lower linear sensitivity range between 0 and 37.22 mmHg in comparison with the other designs that exhibited a linear sensitivity range of 0–41.87 mmHg and 0–46.52 mmHg for toroidal and rectangular designs, respectively. From the previous results, it can be noted that each SSMS-IS design exhibits different levels of linear sensitivity with regard to the external pressure.

Moreover, from Figure 15b, Figure 16b and Figure 17b, there are different profiles of the drift performance showing how the design can influence the drift performance. The toroidal and semi-toroidal designs showed approximately 35% resistance variation, while the rectangular design showed approximately less than 10% resistance variation and also a stable drift by a defined time. Therefore, the low level of drift for the rectangular geometry indicates a system that demands less complexity of calibration and less variance, an essential characteristic for the implementation of filters. 

Furthermore, shown in Figure 18a, Figure 19a and Figure 20a are the different levels of sensitivity for internal pressure tests with regard to the percentage of linear variation, linear range, and the conditions of saturation for each design tested in the same conditions. The toroidal and semi-toroidal designs showed a linear sensitivity variation of approximately 45% and 55%, respectively, while the rectangular design showed a linear sensitivity variation of around 85% for the same range of internal pressure of 0–477.81 mmHg. Regarding the linear range for internal pressures, it can be noted that only semi-toroidal design exhibited a low linear range of 0–97.06 mmHg. This result is directly connected with the saturation presented for pressures above 97.06 mmHg. On the other hand, there is no noted saturation in the rectangular and toroidal designs for the same internal pressure range of 0–477.81 mmHg. Accordingly, the results noted that each SSMS-IS design performs different levels of linear sensitivity regarding the internal pressure.

Also, Figure 18b, Figure 19b and Figure 20b show different profiles of the durability parameter studied for each design of SSMS-IS. However, for all designs, a good response of endurance and system robustness could be noted once 3000 cycles of cyclic loading are a achieved. 

Moreover, Table 2 shows that the linear internal sensitivity range is higher than the linear external sensitivity range, indicating that the SSMS-IS can detect a major range of pressure in the actuated state for all studied designs. Additionally, it is noted from Table 2 that the linear internal sensitivity percentage variation is larger in comparison with the linear external sensitivity percentage variation, showing that the SSMS-IS is more sensible for internal pressures for all studied designs. 

Table 2 and the previous pieces of evidence show that the rectangular design shows the highest values for the investigated parameters: linear external sensitivity percentage of variation, linear external sensitivity range, linear internal sensitivity percentage of variation, and linear internal sensitivity range. Then, this indicates that, since the rectangular design has a mechanical elasticity with fewer constraints, it achieves a better deformability performance in its system in comparison to the other studied designs. For this reason, the rectangular design demands low values of external and internal pressures to change the electrolyte’s area between the electrodes and, consequently, the resistance values of the SSMS-IS, as discussed in [27].

**Figure 23:**
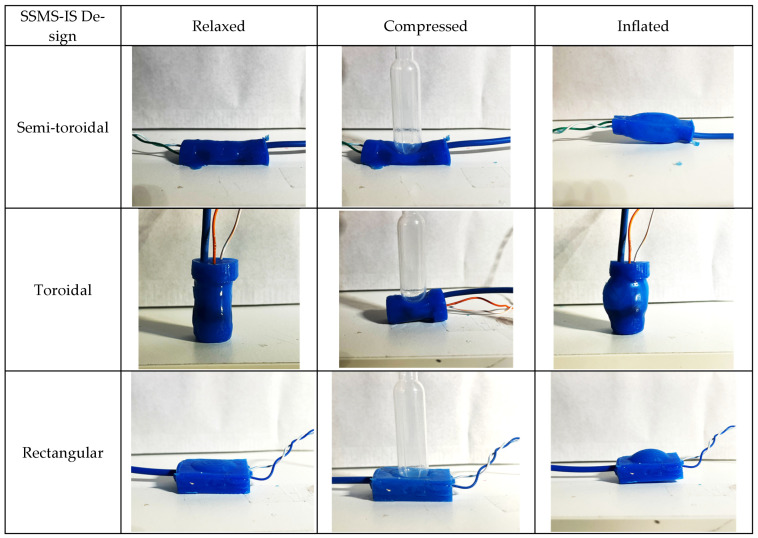
Mechanical deformation for each system design (toroidal, semi-toroidal, and rectangular) for each state of the system: relaxed, compressed, and inflated. The differences in the mechanical elasticity constraints for each SSMS-IS design can be noticed during the states of expansion and compression.

Therefore, the performance results show that it is possible to have performance deviations for different SSMS-IS design projects. The difference in the relationship between stress–strain for each design changes the stiffness of each system. As shown in Figure 23, the differences in the mechanical elasticity constraints for each SSMS-IS design can be noticed during the states of expansion and compression, as discussed in [28]. 

From the test results, it can be concluded that the SSMS-IS can perform a morphologic computation in the soft robot body for the same test conditions. 

The robot interface can morphologically compute different levels of the external forces applied over the robot body based on the resistance change. From Figure 22 and Table 3, it was observed that in the response to load 1, the variance in the resistance for SSMS-IS#1 was 0.0051, while for loads 2 and 3, it was 0.0005 and 0.0010, respectively, representing a variance reduction of 90.19% and 80.39%, respectively, for the same robot state, i.e., relaxed state. The same was observed for the robot actuated state, for load 1, the variance in the resistance for SSMS-IS#1 was 0.0169, while for loads 2 and 3, it was 0.0092 and 0.0086, respectively, indicating a variance reduction of 45.56% and 49.11%, respectively. Based on these variance changes in the SSMS-IS resistance for both robot states, relaxed and actuated, it is observed that the robot can morphologically compute different levels of external forces. 

Also, depending on the robot’s state, it can respond morphologically differently to the same input load applied to its body. Figure 22 and Table 3 show the variance’s resistance for each SSMS-IS for each level of load applied in the robot shield. Also, it shows the percentage of variance difference from the relaxed state to the actuated state. It is noticed from Table 3 that there was a significant reduction in the variance of the SSMS-IS resistance during the actuated state, indicating a reduction in the level of the external load impact over the shield. The variance difference from the relaxed to the actuated state for the SSMS-IS#2 with regard to load 1, load 2, and load 3 was −95.90%, −84.78%, and −70.93%, while for the SSMS-IS#1 with regard to load1, load 2, and load 3, it was −88.75%, +60%, and 0%. Then, SSMS-IS#2 showed greater damping in comparison to SSMS-IS#1, implying that the direction of the external forces might be applied more for a specific side of the robot once the setup of tests cannot guarantee high accuracy for symmetry concerning the shield sides. However, a reduction of more than 70% in the resistance of variance on one side of the shield can be observed, indicating a reduction in the external force intensity applied over the shield. These results indicate that the robot’s morphology can respond in sensory-motor coordination as an intelligent damper. When the SSMS-IS is in the inflated state, the system works as a damper, reducing the impact on the system and consequently reducing the external force variation monitored by SSMS-IS. Otherwise, when the robot is in a relaxed state, it can compute morphologically. 

Some avenues of overcoming limitations of the experimental method are as follows: the influence of the electrolyte degradation in the SSMS-IS response for each type of geometry could be studied; more different geometries of SSMS-IS could have been compared; a larger number of samples could be assessed to improve the accuracy of the results; and other performance parameters for the SSMS-IS could be considered. 

The robot combined with more SSMS-ISs along its shield or the use of another type of sensor, such as gyro, sonar, etc. can improve the precision of the perception estimation as mentioned in Figure 1. Further, a sensor fusion algorithm could be implemented to refine the perception of the robot [29]. However, this was not in the scope of this study. The study results shows that the application of the SSMS-IS for robot systems can have a significant potential application in several areas such as the protection interface between the human body and environmental threats. 

Moreover, robots based on morphologic computation might benefit from using the SSMS-IS once their characteristics such as sensing and actuation are integrated into a unique component to improve the performance in threat scenarios, where fast coordination among perception, control, and actuation is required to guarantee a fast agent response in the environment, as discussed in [20]. Robots using the SSMS-IS can use their actuation property for their locomotion. Once the sensor actuation can achieve different levels of expansion, the robot can be able to walk in different types of terrains or improve the mechanical energy of the system. Also, the locomotion can be monitored simultaneously from the same component, since in the SSMS-IS, the actuation and sensing characteristics are integrated, as discussed in [24]. Moreover, robot prosthetics using soft materials can benefit from using the SSMS-IS. For example, the sensor might be positioned in the robot grip to measure the external pressure from the environment and improve its force control. Moreover, leg prostheses with this sensor can detect and monitor the locomotion of the agent and support the rehabilitation treatment. In addition, their actuation can compensate for wrong walking patterns, helping in physical therapy corrections, as discussed in [25]. For soft robots, sensors with a low level of drift are desired to avoid the high complexity of calibration of the sensor. Also, the sensors need to be physically durable. In summary, for sensory-motor applications in the mentioned bioinspired shield for expansion and compression to detect obstacles and actuation in the environment, the sensibility of the tested soft sensory-motor system based on ionic solution is appropriate. In this regard, for the current robot, the rectangular geometry fits the requirements of the project as it presents high sensibility, stable drift, and durability. 

## 5. Conclusions

This paper has presented a method for the fabrication and performance evaluation of three soft sensory-motor system designs based on ionic solution. The results showed that it is possible to have performance deviations for different SSMS-IS design projects, and this is a particularly critical aspect to consider because an inappropriate SSMS-IS design can result in low performance of a system. Therefore, the performance results show that the rectangular design exhibits the highest performance values for the investigated parameters: high sensitivity, stable drift, and durability. The SSMS-IS is more sensible for internal pressures for all studied designs. It has evaluated the usability and effectiveness of the SSMS-IS design for soft robotics applications, allowing the robot to adapt its morphology online for environment variability, thereby responding to external stimuli from the environment with different levels of stiffness and damping.

Robots based on morphologic computation might benefit from using the SSMS-IS once their characteristics such as sensing and actuation are integrated into a unique component to improve the performance in threat scenarios where a fast coordination among perception, control, and actuation is required. The application of the SSMS-IS for robot systems can have numerous potential applications in several areas such as the protection interface between the human body and environmental threats, morphologic computation, locomotion, robot prosthetics, and rehabilitation treatment. We are excited to see our methodology being deployed in other application domains in the future.

## Figures and Tables

**Table 1:** States of the robot body.

Robot Body State	SSMS-IS#1	SSMS-IS#2
State 1	Disabled	Disabled
State 2	Disabled	Enabled
State 3	Enabled	Disabled
State 4	Enabled	Enabled

**Table 2:** Tests performance results for different designs of SSMS-IS.

Test Performance	Toroidal	Semi-Toroidal	Rectangular
Linear external sensitivity % variation	~20%	~35%	~45%
Linear external sensitivity range	0–41.87 mmHg	0–27.91 mmHg	0–46.52 mmHg
Linear internal sensitivity % variation	~45%	~55%	~85%
Linear internal sensitivity range	0–477.81 mmHg	0–97.06 mmHg	0–477.81 mmHg
Drift	~35%	~35%	~10%
Durability	3000 cycles	3000 cycles	3000 cycles

**Table 3:** Variance and median for each SSMS-IS in the robot for different states and over different external loads.

			Variance (σ^2^)		Median	
	Load	Force	Relaxed	Actuated	Relaxed	Actuated
SSMS-IS#1	1	1.8 N	0.0051	5.7350 × 10^−4^	0.29435	0.48666
	2	2.4 N	0.0005	0.0008	0.12351	0.46279
	3	2.9 N	0.0010	0.0010	0.07426	0.43887
SSMS-IS#2	1	1.8 N	0.0169	6.9240 × 10^−4^	0.46279	0.53425
	2	2.4 N	0.0092	0.0014	0.46279	0.53425
	3	2.9 N	0.0086	0.0025	0.46279	0.51048

## Data Availability

The raw data supporting the conclusions of this article will be made available by the authors on request.

## Nomenclature

Symbol	Description	Unit
$P_{ext}$	External pressure	N·m^−2^
$P_{int}$	Internal pressure	N·m^−2^
L	Length between electrodes	mm
A	Area of the electrolyte between electrodes	mm^2^
R	Electric resistance	Ω
ρ	Electrical resistivity	-
V	Voltage between electrodes	kg·m^2^·s^−3^·A^−1^
$F_{ext}$	External force	N
Abbreviation	Description	
SSMS-IS	Soft sensory-motor system based on ionic solution	-
